# 

**Published:** 1890-07

**Authors:** 


CONTENTS:

Article I.—Impressions and Impression-Taking
By Mr. E Bull. - - - - - - 97

" II.—Observations on the Structure and Development of Ovarian Teeth. By T. Charters White and J. Bland Sutton, F.R.C.S. - - - - 107

" III.—Gold and Tin in saving Teeth. By C. R. Butler. - 116

" IV.—The care of the Vulcanizer. By G. B. Snow, D. D. S. 121

" V.—Notes on a case of Syphilitic cleft Palate, with its Treatment. By E. Lloyd Williams, L. R. C. P. - 125

" VI.—Root-Filling in its Relation to Disease.
By Rodrigues Ottolengui, M. D. S. - - - 128

COMMUNICATIONS.

American Dental Association. - - - - - 140

National Association of Dental Examiners. - - - - 140

EDITORIAL.

Acromegaly. - - - - - - - - - - 141

Breathing through the Mouth a cause of Decay of the Teeth. - 142

MONTHLY SUMMARY.

A Cheshire Farmer and his False teeth. - - - - - - 143

Dentistry in 1892. - - - - - - - - 144

